# Autophagy Inhibits Grass Carp Reovirus (GCRV) Replication and Protects *Ctenopharyngodon idella* Kidney (CIK) Cells from Excessive Inflammatory Responses after GCRV Infection

**DOI:** 10.3390/biom10091296

**Published:** 2020-09-08

**Authors:** Pengfei Chu, Libo He, Rong Huang, Lanjie Liao, Yongming Li, Zuoyan Zhu, Wei Hu, Yaping Wang

**Affiliations:** 1 State Key Laboratory of Freshwater Ecology and Biotechnology, Institute of Hydrobiology, Chinese Academy of Sciences, Wuhan 430072, China; chupengfei17@163.com (P.C.); huangrong@ihb.ac.cn (R.H.); liaolj@ihb.ac.cn (L.L.); liym@ihb.ac.cn (Y.L.); zyzhu@ihb.ac.cn (Z.Z.); huwei@ihb.ac.cn (W.H.); 2 University of Chinese Academy of Sciences, Beijing 100049, China; 3 College of Animal Science and Technology, Yangzhou University, Yangzhou 225009, China; 4 Innovative Academy of Seed Design, Chinese Academy of Sciences, Beijing 100101, China

**Keywords:** autophagy, grass carp, grass carp reovirus, inflammatory responses

## Abstract

Autophagy is an essential and highly conserved process in mammals, which is critical to maintaining physiological homeostasis, including cell growth, development, repair, and survival. However, the understanding of autophagy in fish virus replication is limited. In this study, we found that grass carp reovirus (GCRV) infection stimulated autophagy in the spleen of grass carp (*Ctenopharyngodon idella*). Moreover, both Western blot (WB) analysis and fluorescent tracer tests showed that GCRV infection induced the enhancement of autophagy activation in *Ctenopharyngodon idella* kidney (CIK) cells. Autophagy inducer rapamycin and autophagy inhibitor 3-MA pretreatment can inhibit and promote the proliferation of GCRV, respectively. In addition, grass carp autophagy-related gene 5 (CiATG5)-induced autophagy, as well as rapamycin, showed effects on GCRV replication in CIK cells. Transcriptome analysis revealed that the total number of differentially expressed genes (DEGs) in CiATG5 overexpression groups was less than that of the control during GCRV infection. Enrichment analysis showed that CiATG5 overexpression induced the enhancement of autophagy, lysosome, phagosome, and apoptosis in the early stage of GCRV infection, which led to the clearance of viruses. In the late stage, steroid biosynthesis, DNA replication, terpenoid backbone biosynthesis, and carbon metabolism were upregulated, which contributed to cell survival. Moreover, signaling pathways involved in the immune response and cell death were downregulated in CiATG5 overexpression groups. Further study showed that CiATG5 repressed the expression of inflammatory response genes, including cytokines and type I interferons. Taken together, the results demonstrate that autophagy represses virus replication and attenuates acute inflammatory responses to protect cells.

## 1. Introduction

Autophagy is a highly conserved process in eukaryotes by which intracellular material is degraded and recycled in response to nutrient starvation or other hostile environments [1,2,3]. More than 40 autophagy-related genes (ATGs) are involved in the regulation of the autophagy process [4,5]. In mammalian cells, nutrient starvation is a classic pathway to activate autophagy. Mammalian target of rapamycin complex 1 (mTORC1) activity is switched off when cells sense insufficient nutrition, and, subsequently, the cells release unc-51-like autophagy activating kinase 1 (ULK1), initiating autophagy signaling [6,7]. Therefore, ULKs are thought to be the upstream components of the autophagy pathway that initiate the downstream ATG conjugate cascades [8]. During the autophagy process, the formation of the ATG16L1 complex (composed of ATG16L1, ATG5, and ATG12) is key; it functions as an E3-like enzyme for another ubiquitin-like system, LC3 [9]. Phosphatidylethanolamine (PE)-conjugated LC3 (LC3-II) is located in the isolation membrane, where the ATG16L1 complex accumulates, while unconjugated LC3 (LC3-I) is evenly distributed in cells [10]. Moreover, LC3-II and LC3-I can also be detected separately by immunoblot; therefore, LC3 is used as a preferred marker for microscopic detection of isolation membranes and autophagosomes [5,11,12].

Accumulating evidence indicates that autophagy is closely related to viral infection and replication in mammals. The induction of autophagy by rapamycin has been shown to increase Japanese encephalitis virus (JEV) production, whereas the inhibition of autophagy by 3-methyladenine (3-MA) reduced viral yields; further studies have shown that JEV replication is related to ATG5 or Beclin 1 expression [13]. Several key autophagy proteins (i.e., Beclin-1, ATG4B, ATG5, and ATG12) are pivotal factors required for the translation of incoming hepatitis C virus (HCV) RNA, but they are not required once the infection is established [14]. The current data support that autophagy impacts the late step of human hepatitis B virus (HBV) replication, increasing HBV production [15]. Inhibition of autophagy suppressed HBV DNA synthesis, suggesting an enhancement of HBV DNA replication by autophagy [16]. Scientists further found that the inhibition of autophagy in ATG5-knockout mice could reduce HBV gene expression and affect the nuclear localization of the HBV core protein [17]. Some RNA viruses even instigate the double-membrane compartments formed during autophagy to provide a small isolated membrane envelope for viral replication, protecting viral RNAs from detection by innate immune sensors and degradation. Although poliovirus lacks a membrane envelope, it can promote the infected cells to reveal double-membrane vesicles that provide scaffolds for viral RNA replication [18]. Further studies have confirmed that the induction of autophagy by rapamycin increases poliovirus replication, and the inhibition of autophagy by silencing key ATGs decreases poliovirus replication [19]. On the other hand, as an indispensable component of the immune system, autophagy targets the invading viruses to degrade and dispose of viral components, viral particles, or even host factors required for viral replication [20]. Thus, autophagy also functions as an essential innate antiviral response. HCV is usually thought to use autophagy for its replication, while some researchers have shown that autophagy suppresses HCV replication via the endoplasmic reticulum (ER) protein SCOTIN. HCV nonstructural 5A (NS5A) protein, which is a critical factor for HCV RNA replication, interacts with the IFN-β-inducible protein SCOTIN, which transports NS5A to autophagosomes for degradation [21]. Previous studies have confirmed that HIV-1 is degraded in an autophagosome-dependent pathway by an autophagy-dependent pathway. Histone deacetylase 6 (HDAC6) mediates autophagy-dependent inhibition of HIV-1 replication by interaction with APOBEC3G [22]. The restriction factor tripartite motif-containing protein 5α (TRIM5α) mediates the maturation of autophagy to engulf HIV-1 to autophagic degradation [23]. It seems autophagy functions as a double-edged sword for antiviral infection.

Although studies that elucidate the autophagy functions involved in viral infection are sufficient in mammals, there are few reports in fish [24]. The first report on fish-virus-induced autophagy was the infectious salmon anemia virus (ISAV) in 2010, which showed that ISAV-induced autophagy and inhibition of autophagy by 3-MA reduced viral production [25]. The infectious spleen necrosis virus (ISKNV) induced autophagy and promoted autophagic flux in the infected cells, and the inhibition of the formation of autophagolysomes promoted the production of the infectious virus [26]. A recent study demonstrated that ATG5-induced autophagy increased the Singapore grouper iridovirus (SGIV) and red-spotted grouper nervous necrosis virus (RGNNV) production in orange-spotted grouper (*Epinephelus coioides*) [27]. However, consistent with the results in mammals, some studies found that autophagy plays an antiviral role in response to viral infection. In turbot (*Scophthalmus maximus*) erythrocytes, autophagy participates in resistance against the viral hemorrhagic septicemia virus (VHSV) [28]. Grass carp (*Ctenopharyngodon idella*) is an important aquaculture species in China. Grass carp hemorrhagic disease caused by grass carp reovirus (GCRV) has brought huge damage to the grass carp industry. However, the functions of autophagy in GCRV infection and replication remain largely uncharacterized. In this study, GCRV-induced autophagy is confirmed in the spleen of grass carp and CIK cells. Moreover, the role of a core autophagy-related gene (CiATG5) is investigated during GCRV infection. Furthermore, the relationship between autophagy and GCRV replication is analyzed. The results provide new evidence of autophagy-restricted virus replication in teleosts.

## 2. Materials and Methods 

### 2.1. Experimental Fish, Cells, and Virus Infection

Grass carp (40 ± 10 g) used in the study were bred and cultivated in the GuanQiao Experimental Station, Institute of Hydrobiology, Chinese Academy of Sciences. For the GCRV infection experiment, grass carp were intraperitoneally injected at a dose of 2% (vol/g) virus fluid to fish quality. The control groups were injected with phosphate buffer saline (PBS) (Gibco, Grand Island, NY, USA) [29]. CIK cells were bought from the China Center for Type Culture Collection, incubated in 28 °C, and maintained in low glucose Dulbecco’s modified Eagle’s medium (DMEM, Gibco, Grand Island, NY, USA) supplemented with 10% fetal bovine serum and 1% (*v*/*v*) penicillin–streptomycin in a humidified atmosphere with 5% CO_2_. For virus infection in cells, CIK cells were plated for 24 h in advance and then infected with GCRV at a multiplicity of infection (MOI) of 1. After 3 h, the virus inoculum was removed and the cells were incubated with new medium (DMEM). The control groups were treated with PBS.

### 2.2. Plasmid Construction, Transfection, and Reagents

Grass carp LC3B was subcloned into pEGFP-N3 (Clontech, Mountain View, CA, USA) to generate GFP-LC3B to detect the formation of autophagy. Grass carp ATG5 was subcloned into pCMV-FLAG, pDsRed2-C1, and pEGFP-N3 (Clontech, Mountain View, CA, USA ) to generate FLAG-ATG5, DsRed-ATG5, and GFP-ATG5 to overexpress ATG5 in CIK cells. The plasmids were constructed as described previously [30,31] Then, these vectors (500 ng/µL) were transfected into CIK cells by Lipofectamine™ 3000 Transfection Reagent (Invitrogen, Carlsbad, CA, USA), according to the manufacturer’s recommendations. Rapamycin, 3-MA, and horseradish peroxidase (HRP)-conjugated goat anti-rabbit IgG were purchased from Sigma (Saint Louis, MS, USA). Rabbit polyclonal anti-LC3B antibody was purchased from Abcam (Cambridge, UK). Rabbit polyclonal SQSTM1/P62 antibody was purchased from Beyotime (Shanghai, China). Lyso-Tracker Red fluorescent probe was purchased from Solarbio (Beijing, China).

### 2.3. RNA Isolation, Library Construction, and Sequencing

CIK cells were sampled at 0, 6, 12, and 24 h after GCRV infection (named GCRV-H0, GCRV-H6, GCRV-H12, and GCRV-H24, respectively; three biological duplicates for each group), rapidly frozen in liquid nitrogen, and total RNA was extracted with Trizol reagent (Invitrogen, Carlsbad, CA, USA). Sequencing libraries were generated using NEBNext^®^ UltraTM RNA Library Prep Kit for Illumina^®^ (NEB, Ipswich, MA, USA) following the manufacturer’s recommendations. Briefly, mRNA was purified from total RNA using poly-T oligo-attached magnetic beads. Fragmentation was carried out using divalent cations under elevated temperature in NEBNext First Strand Synthesis Reaction Buffer. First-strand cDNA was synthesized using random hexamer primer. Second strand cDNA synthesis was subsequently performed using DNA Polymerase I and RNase H. After adenylation of 3′ ends of DNA fragments, NEBNext adaptors with hairpin loop structure were ligated to prepare for hybridization. In order to select cDNA fragments of preferentially 250~300 bp in length, the library fragments were purified with the AMPure XP system (Beckman Coulter, Beverly, CA, USA). Then, 3 µL USER Enzyme (NEB, Ipswich, MA, USA) was used with size-selected, adaptor-ligated cDNA at 37 °C for 15 min, followed by 5 min at 95 °C before PCR. Then, PCR was performed with Phusion High-Fidelity DNA polymerase, Universal PCR primers, and Index (X) primer. At last, PCR products were purified (AMPure XP system), and library quality was assessed on the Agilent Bioanalyzer 2100 system. The library preparations were sequenced on an Illumina Novaseq platform, and 150 bp paired-end reads were generated.

Additionally, CIK cells were transfected with FLAG-ATG5 or empty vectors for 24 h, as described above [32], followed by GCRV infection. Cells were sampled at 0, 6, 12, and 24 h after GCRV infection (named ATG5-H0, ATG5-H6, ATG5-H12, ATG5-H24, VT-HO, VT-H6, VT-H12, and VT-H24, respectively; three biological duplicates for each group). RNA isolation, library construction, and sequencing methods of these samples were the same as above.

### 2.4. Differential Expression Analysis of Transcriptome Sequencing

The grass carp genome (accession number: PRJEB5920) was used as a reference genome for further analysis. DESeq2 R package (1.16.1) was used to perform differential expression analysis [33]. The resulting *p*-values were adjusted using Benjamini and Hochberg’s approach for controlling the false discovery rate. Genes with an adjusted *p*-value <0.05 (padj < 0.05), found by DESeq2, were assigned as differentially expressed genes (DEGs).

Gene Ontology (GO) classification, including GO-BP (biological process), GO-MF (molecular function), and GO-CC (cellular component), implemented by the clusterProfiler R package was applied to uncover the functions of intersecting genes [34,35]. GO terms with a corrected *p*-value of less than 0.05 were considered significantly enriched by DEGs. The Kyoto Encyclopedia of Genes and Genomes (KEGG) database is used for understanding high-level functional information in biological systems from molecules, cells, organisms, and ecosystems, and it is particularly powerful for large-scale molecular datasets generated by genome sequencing and other high-throughput experimental approaches [36]. ClusterProfiler R package was used to test the statistical enrichment of DEGs in KEGG pathways [34,35]. KEGG terms with corrected *p*-values of less than 0.05 were considered significant. Gene Set Enrichment Analysis (GSEA) is a computational approach to determine if a predefined gene set can show a significant, consistent difference between two biological states. In the study, we used the local version of the GSEA analysis tool http://www.broadinstitute.org/gsea/index.jsp. GO, KEGG, Reactome, DO, and DisGeNET data sets were used for GSEA independently.

### 2.5. Gene Expression Analysis

Quantitative real-time polymerase chain reaction (qRT-PCR) was performed to detect the expression levels of GCRV-related genes, autophagy-related genes, and immunity-related genes. All primers used in the study were designed by software Primer Premier 5.0 and are listed in Table S5. The relative expression ratio of the selected gene vs. β-actin (reference gene) was calculated using the 2^−∆∆Ct^ method. Reactions of SYBR Green were performed in a 20-µL volume containing 10 µL of 2× SYBR^®^Green Realtime PCR Master Mix (Toyobo, Osaka, Japan), 1 µL of each forward and reverse primer (10 µM), 7 µL of water, and 1 µL of diluted cDNA (100 ng/µL). All experiments were performed in triplicate.

### 2.6. Immunohistochemistry and WB

The spleen of GCRV- or PBS-infected grass carp were sampled, formalin-fixed, and embedded in paraffin. The paraffin sections were then incubated with primary antibodies against LC3B at 4 °C for 12 h, followed by incubation with HRP-conjugated goat anti-rabbit IgG at room temperature for 2 h. The sections were detected with 3-3′-diaminobenzidine (DAB) according to the manufacturer’s instructions (Sangon Biotech, Shanghai, China). After staining the cell nucleus with hematoxylin solution, the sections were observed under a microscope (Zeiss, Jena, Germany).

For WB analysis, CIK cells were lysed in RIPA buffer on ice for 30 min. Then, the lysates were collected and centrifuged at 12,000× *g* at 4 °C for 10 min. Proteins were separated by 15% SDS-PAGE and transferred to a polyvinylidene difluoride membrane (PVDF; Millipore, Burlington, MA, USA). PVDFs were washed three times with Tris-Buffered Saline Tween-20 (TBST) (Kangwei, Beijing, China) and incubated with 5% nonfat milk powder at room temperature for 1 h, followed by incubation with anti-LC3B or anti-P62 antibody at 4 °C for 12 h. After washing in TBST, PVDF membranes were incubated with HRP-conjugated goat anti-rabbit IgG at room temperature for 1 h. For detection, Immobilon Western Chemiluminescent HRP Substrate (Millipore, Burlington, MA, USA) and Fujifilm LAS4000 mini-luminescent image analyzer were used to detect the blot according to the manufacturer’s instructions. Additionally, the relative protein levels were quantified by Image J software.

### 2.7. Fluorescence Microscopy

CIK cells were transfected with GFP-LC3B vectors for 6 h, then infected with GCRV at an MOI of 1 or PBS (control). At 6, 12, and 24 h after GCRV infection, cells were fixed with 4% paraformaldehyde and stained with Hoechst 33342 (Beyotime, Shanghai, China). Then, CIK cells were observed using the confocal system (Leica SP8, Weztlar, Germany) and a 63× oil immersion objective lens. For lysosome detection, cells were infected with GCRV for 23 h and then incubated with lyso-tracker for 1 h before fixing with 4% paraformaldehyde. Moreover, one group of CIK cells were cotransfected with GFP-LC3B and DsRed-ATG5 or DsRed vectors for 23 h, and another group of CIK cells was transfected with GFP-ATG5 or empty vectors for 23 h, followed by incubation with lyso-tracker for 1 h, and then all cell samples were similarly treated with the former method.

### 2.8. Statistical Analysis

The statistical results (expressed as mean ± standard deviation) were analyzed by one-way analysis of variance, followed by Dunnett’s test for multiple comparisons using SPSS Statistics 19 software. *p* < 0.05 was considered to be statistically significant. All experiments were repeated at least three times.

### 2.9. Ethics Statements

Animal welfare and experimental procedures were carried out in accordance with the Guide for the Care and Use of Laboratory Animals (Ministry of Science and Technology of China, 2006), and the protocol was approved by the committee of the Institute of Hydrobiology, Chinese Academy of Sciences (CAS). All surgery was performed under eugenol anesthesia, and all efforts were made to minimize animal suffering.

## 3. Results

### 3.1. GCRV Infection Stimulated Autophagy in the Spleen of Grass Carp

**Figure 1:**
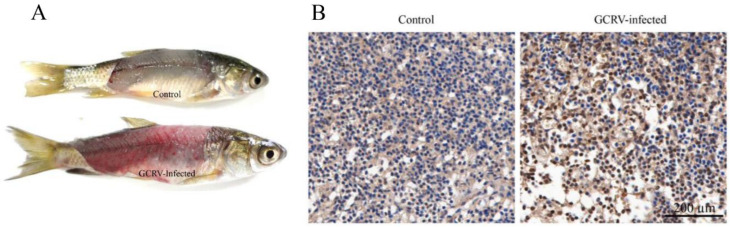
Grass carp reovirus (GCRV) infection induced autophagy in the spleen of grass carp. (**A**) GCRV infection caused hemorrhagic symptoms of grass carp. (**B**) The spleens of GCRV- or PBS-infected fish were sampled for immunohistochemistry analysis. Cells stained blue are normal, and the cells labeled by anti-LC3B antibody are brown (scale bar, 200 µm).

To determine whether autophagy was activated by GCRV infection, grass carp were infected with GCRV. As showed in Figure 1A, compared with the control, GCRV infection caused obvious hemorrhagic symptoms in the muscle of infected fish. Furthermore, the spleens of grass carp were sampled for immunohistochemistry in order to detect whether GCRV induced autophagy. As showed in Figure 1B, compared with the control, the proportion of brown cells (indicating autophagy) in GCRV-infected spleen increased significantly. Therefore, the result revealed that GCRV infection caused significant autophagy in the spleen. 

### 3.2. GCRV Infection Triggered Autophagy in CIK Cells

**Figure 2:**
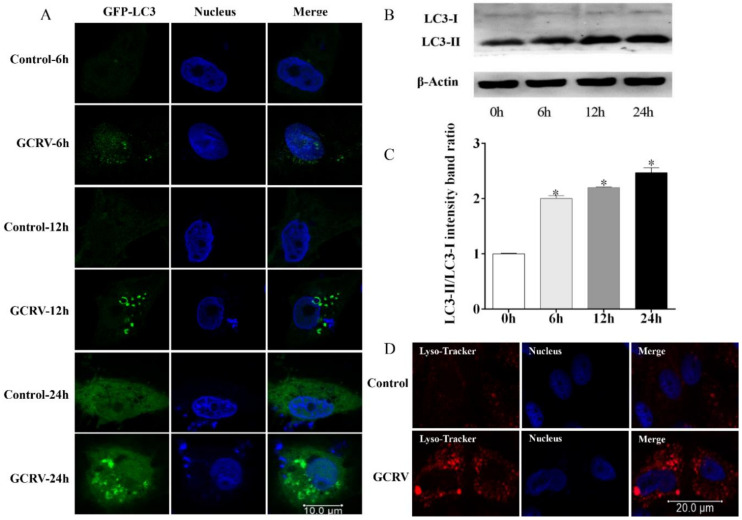
GCRV infection triggered autophagy in *Ctenopharyngodon idella* kidney (CIK) cells. (**A**) GCRV infection was performed after CIK cells were transfected with GFP–LC3B vectors for 6 h. Cells were sampled at 6, 12, and 24 h after GCRV infection, and then the cells were fixed and stained for confocal microscopy (Leica SP8, Weztlar, Germany; scale bar, 10 μm). (**B**,**C**) Cells were sampled at 0, 6, 12, 24 h after GCRV infection, and WB analysis was used to measure the autophagic levels. The relative intensity of LC3-II to LC3-I was quantified by Image J software. Significant differences (*p* < 0.05) in relation to the control group were indicated with an asterisk (*). (**D**) CIK cells were infected with GCRV for 23 h, followed by incubation with lyso-tracker for 1 h, and then cells were treated the same way as above (scale bar, 20 μm).

To further investigate the functions of autophagy during GCRV infection, CIK cells were used as a model to clarify whether GCRV could induce autophagy in vitro. As autophagy matures, LC3-I is conjugated with phosphatidylethanolamine to form LC3-II, and the two forms of LC3 can also be detected separately by immunoblot [11]. Moreover, LC3-II is located in the isolation membrane, while LC3-I is evenly distributed in cells [10]. Therefore, LC3 is used as a faithful marker of autophagy activation for both immunoblot and microscopic detection [12]. Compared with the control, GCRV infection significantly induced autophagy, as evidenced by GFP–LC3-II-labeled autophagosomes (Figure 2A). The GFP–LC3-II puncta, which are called autophagosomes, appeared in the cytoplasm as early as 6 h post-GCRV infection, and the puncta accumulated over time in the course of the GCRV infection (Figure 2A). Further, WB analysis was used to assess the formation of endogenous LC3-II. The results showed that GCRV infection significantly enhanced LC3-II expression and the LC3-II/LC3-I ratio (Figure 2B,C). Moreover, lysosomes, which are always accompanied by autophagy activation, were accumulated after GCRV infection (Figure 2D). Collectively, these findings suggest that GCRV infection triggers autophagy in CIK cells. 

### 3.3. Autophagy Inhibited GCRV Replication in CIK Cells

**Figure 3:**
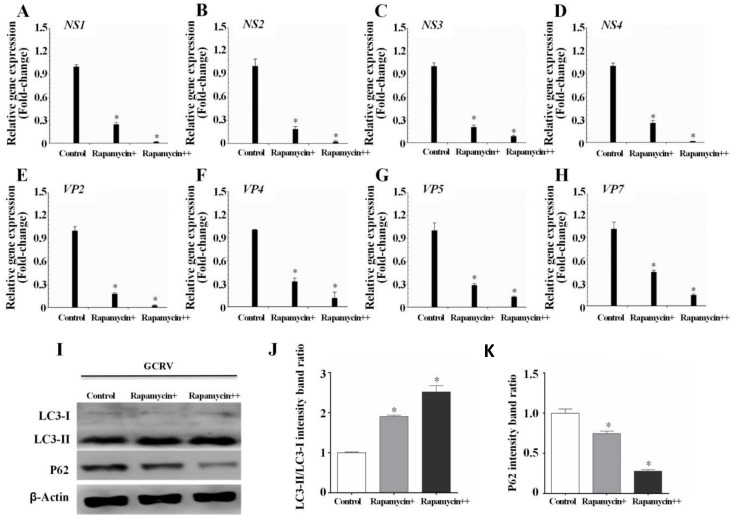
Rapamycin-induced autophagy restricted GCRV replication. (**A**–**H**) CIK cells were incubated with 0, 50, and 100 nM rapamycin for 6 h, followed by GCRV infection. After 24 h, cells were sampled to measure the expression of NS and VP genes of GCRV, and one-way ANOVA was used to evaluate the variability between treatment groups. (**I**–**K**) WB analysis was used to detect the autophagic levels of CIK cells treated with rapamycin. The relative intensity of LC3-II to LC3-I and P62 was quantified by Image J software. Significant differences (*p* < 0.05) in relation to the control group were indicated with an asterisk (*).

**Figure 4:**
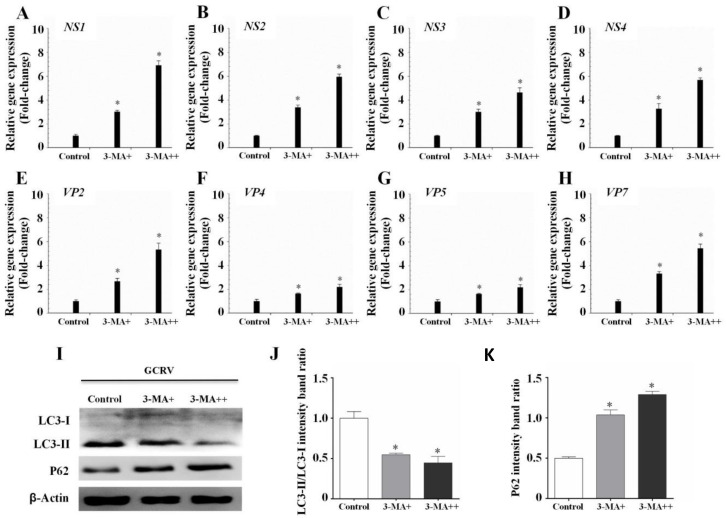
3-MA-inhibited autophagy increased GCRV replication. (**A**–**H**) CIK cells were incubated with 0, 5, and 10 mM 3-MA for 6 h, followed by GCRV infection. After 24 h, cells were sampled to measure the expression of NS and VP genes of GCRV and one-way ANOVA was used to evaluate the variability between treatment groups (*p* < 0.05). (**I**–**K**) WB analysis was used to detect the autophagic levels of CIK cells treated with 3-MA. The relative intensity of LC3-II to LC3-I and P62 was quantified by Image J software. Significant differences (*p* < 0.05) in relation to the control group were indicated with an asterisk (*).

Rapamycin and 3-MA are classic autophagy inducers and inhibitors, respectively [25,27]. To determine the precise role of autophagy in regulating GCRV infection, CIK cells were incubated with different doses of rapamycin or 3-MA, followed by GCRV infection. LC3B and P62 (P62, also named SQSTM1, is an autophagic substrate and its concentration is inversely proportional to autophagic levels [37]) were used to estimate the autophagic levels of CIK cells, and qPCR was used to determine viral replication. WB analysis showed that rapamycin pretreatment significantly enhanced LC3-II expression and the LC3-II/LC3-I ratio, but decreased P62 levels (Figure 3I–K), whereas the 3-MA treatment presented opposite trends (Figure 4I–K), which suggested that rapamycin significantly enhanced autophagic levels while 3-MA decreased autophagic levels. The expression of GCRV genes, including NS1-4, VP2, VP4, VP5, and VP7, were all inhibited in rapamycin-pretreated cells (Figure 3A–H), whereas viral gene expression was increased when autophagy was inhibited by 3-MA (Figure 4A–H). Additionally, the effect of rapamycin and 3-MA on virus production was in direct proportion to the doses. These results reveal that autophagy indeed restricts GCRV replication.

### 3.4. CiATG5 Promoted Autophagy in CIK Cells

**Figure 5:**
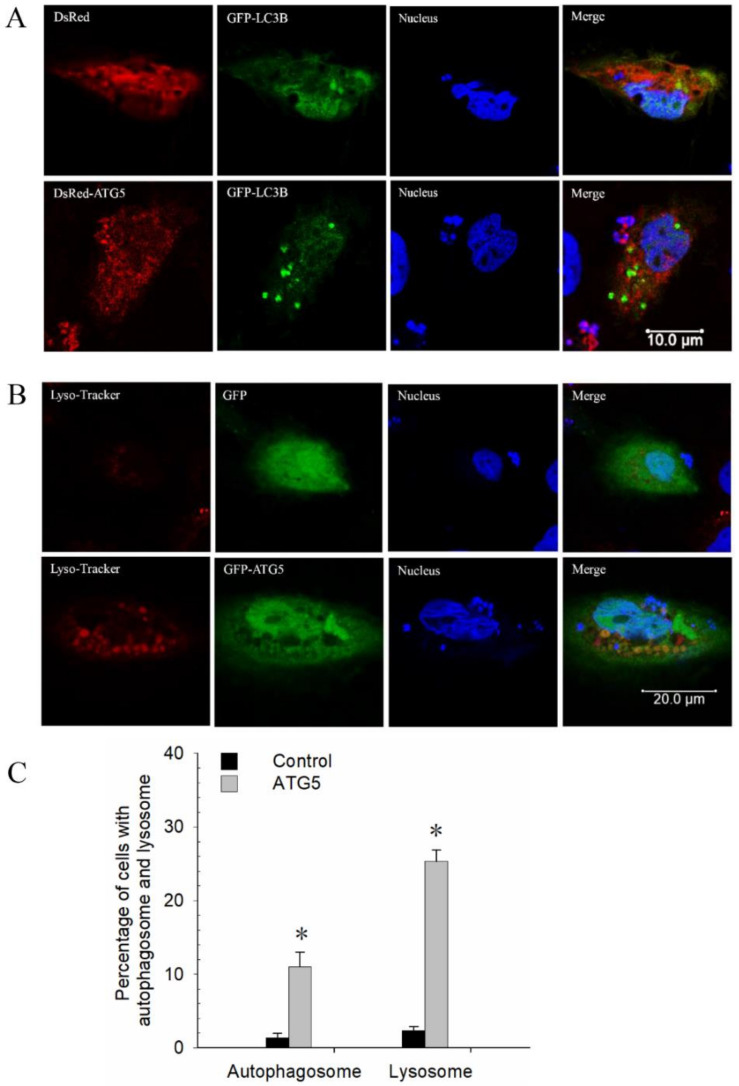
CiATG5 promoted autophagy in CIK cells. (**A**) Cells were cotransfected with GFP–LC3B and DsRed–ATG5 or DsRed (as control group). After 24 h, the cells were fixed and stained for confocal microscopy (Leica SP8, Weztlar, Germany; scale bar, 10 μm). Green fluorescence shows the distribution of LC3B, red fluorescence shows the distribution of CiATG5, and blue fluorescence shows the nucleus stained with Hoechst 33342. (**B**) Cells were transfected with GFP–ATG5 or GFP (as control group) for 23 h, followed by incubation with lyso-tracker for 1 h, then cells were treated the same way as above (scale bar, 20 μm). (**C**) Quantifying the percentage of cells with autophagosomes and lysosomes. Autophagosomes or lysosomes were quantified by the number of cells with at least five positive puncta per cell, accounting for the positive cells. Significant differences (*p* < 0.05) in relation to the control group were indicated with an asterisk (*).

In mammals, ATG5 plays a key role in autophagy. Grass carp ATG5 (CiATG5) was reported to be involved in the immune response after GCRV infection [38]. To further assess the precise role of CiATG5, DsRed-ATG5 was constructed and cotransfected with GFP–LC3B into CIK cells. As showed in Figure 5A, red fluorescent signals showed CiATG5 was located in both cytoplasm and nucleus, and the green puncta labeled by LC3-II were observed in CiATG5-overexpressed cells, while there were no puncta in control cells. Moreover, compared with the control, CiATG5 significantly increased autophagy-related lysosomal activities (Figure 5B,C). These results indicate that CiATG5 promotes autophagy.

### 3.5. CiATG5 Overexpression Inhibited GCRV Replication

**Figure 6:**
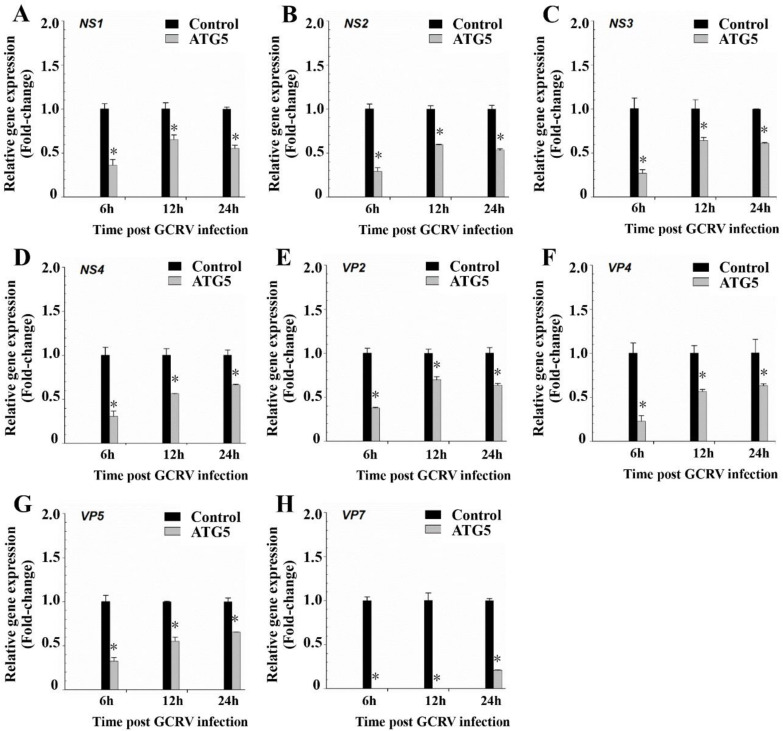
CiATG5 inhibited GCRV replication. CIK cells were transfected with CiATG5 or empty vectors for 24 h, followed by GCRV infection. The cells sampled at 6, 12, and 24 h after GCRV infection were used to measure the expression of NS and VP genes of GCRV, (**A**) NS1, (**B**) NS2, (**C**) NS3, (**D**) NS4, (**E**) VP2, (**F**) VP4, (**G**) VP5, and (**H**) VP7. One-way ANOVA was used to evaluate the variability between treatment groups and significant differences (*p* < 0.05) in relation to the control group were indicated with an asterisk (*).

To explore the influence of CiATG5 on GCRV infection, CIK cells were transfected with CiATG5 for 24 h, followed by GCRV infection. Cells were harvested at 6, 12, and 24 h postinfection, and viral replication was assessed by measuring the expression levels of structural proteins genes (NS1, NS2, NS3, and NS4) and nonstructural proteins genes (VP2, VP4, VP5, and VP7). Compared with the control, the expression levels of the eight detected genes, including NS1-4, VP2, 4, 5, and 7, were all significantly decreased at all detected times (Figure 6A–H), which was consistent with the former results in rapamycin-pretreated cells. The results demonstrate that CiATG5 inhibits GCRV replication.

### 3.6. GCRV Infection Caused Cell Damage and Acute Inflammatory Response

Cell death caused by the rapid proliferation of GCRV after invasion has been fully verified [39,40] (Figure S1). To understand the pathogenesis mechanism, we performed RNA-seq analysis on the samples collected from four time-points after GCRV infection (0, 6, 12, and 24 h postinfection). Three duplicates of each sample were processed, yielding a total of 12 libraries. The sequencing data in this study have been deposited in the Sequence Read Archive (SRA) at the National Center for Biotechnology Information (NCBI; accession number: PRJNA597582 and PRJNA597542). Data from 6, 12, and 24 h postinfection were compared with that at 0 h to identify DEGs. GO enrichment analysis and KEGG pathway analysis were performed to investigate the possible role of DEGs, and the top GO terms and KEGG pathways are listed in Table 1 and Table S3, respectively. The results showed that lysosome and autophagy pathways were inhibited in the early stage of GCRV infection (6 h), but they were upregulated in the late stage (24 h). Additionally, cell components, including cytoplasmic parts, ribosomes, nonmembrane-bound organelles, and mitochondrial membranes, and metabolic pathways, including oxidative phosphorylation, carbon metabolism, propanoate metabolism, and fatty acid degradation and metabolism, were all downregulated in the late stage of GCRV infection (24 h), suggesting that GCRV infection reduces cell metabolism and causes cell damage. However, the stress response involved in response to oxidative stress, the regulation of response to stimulus, the regulation of signal transduction cell communication, and the PRR pathways involved in C-type lectin receptor, RIG-I-like receptor, Toll-like receptor, and NOD-like receptor signaling pathways were upregulated, implying that the acute immune response and inflammatory response were induced.

### 3.7. CiATG5 Weakened Host Inflammation Response to GCRV Invasion

**Figure 7:**
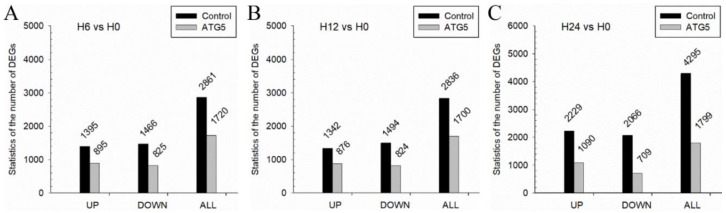
The number of DEGs after GCRV infection. CIK cells were transfected with CiATG5 or empty vectors for 24 h, followed by GCRV infection. The cells sampled at 0, 6, 12, and 24 h after GCRV infection were analyzed by transcriptome sequencing, and samples collected from the CiATG5- or empty-vector-transfected groups at 6 (**A**), 12 (**B**), and 24 h (**C**) postinfection were compared with samples from the 0 h group to identify DEGs. The number of DEGs was counted.

**Figure 8:**
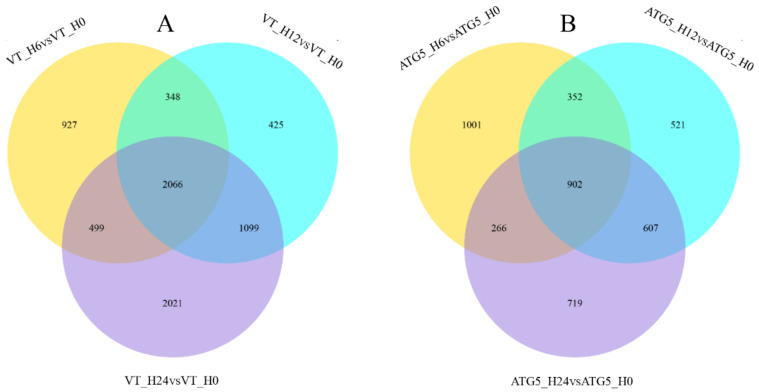
Venn diagrams of DEGs among different groups. Overlapping regions represent the common DEGs in groups. (**A**) Venn diagram of DEGs among the control groups. (**B**) Venn diagram of DEGs among the ATG5 overexpression groups.

The former results showed that both CiATG5-induced and rapamycin-induced autophagy inhibited GCRV replication (Figure 3 and Figure 6). To understand this inhibition mechanism, cells transfected with CiATG5 or empty vectors were collected at four time-points after GCRV infection (0, 6, 12, and 24 h) to perform RNA-seq analysis. Three duplicates of each sample were processed, yielding a total of 24 libraries. The sequencing data have been deposited in the SRA at the NCBI (accession number: PRJNA597622, PRJNA597620, PRJNA597618, and PRJNA597579). Firstly, intragroup comparisons were performed. In each group, data from 6, 12, and 24 h postinfection were compared with that of 0 h to identify DEGs. In general, the total number of DEGs in the control was higher than that of CiATG5 overexpression cells (Figure 7A–C), suggesting the few molecular events had occurred in CiATG5 overexpression cells. Further, these DEGs were subjected to Venn diagram analysis (Figure 8), which identified 2066 common DEGs in the control groups (Figure 2A), while only 902 common DEGs were identified in the CiATG5 overexpressed groups (Figure 2B).

Additionally, intergroup comparisons were carried out to reveal the role of autophagy induced by CiATG5 in response to GCRV infection. In detail, data from CiATG5 overexpression cells were compared with that of the control at the corresponding time points (ATG5–H0 vs. VT–H0, ATG5–H6 vs. VT–H6, ATG5–H12 vs. VT–H12, and ATG5–H24 vs. VT–H24). GO enrichment analysis and KEGG pathway analysis were performed to investigate the possible role of DEGs, and the top GO terms and KEGG pathways are listed in Table 2 and Table S4.

**Figure 9:**
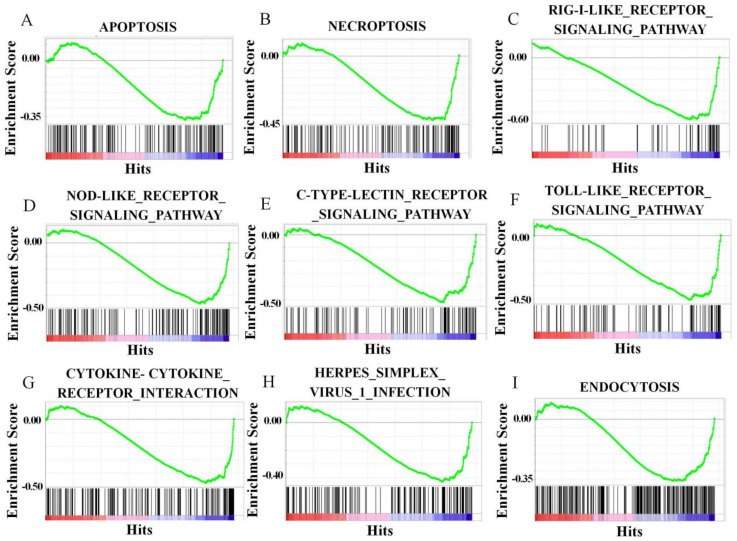
Gene set enrichment analysis (GSEA) of the downregulated genes. The downregulated genes were mainly enriched in apoptosis (**A**), necroptosis (**B**), the RIG-I-like receptor signaling pathway (**C**), the NOD-like receptor signaling pathway (**D**), the C-type lectin receptor signaling pathway (**E**), the Toll-like receptor signaling pathway (**F**), the cytokine-cytokine receptor interaction (**G**), Herpes simplex virus 1 infection (**H**), and endocytosis (**I**).

**Figure 10:**
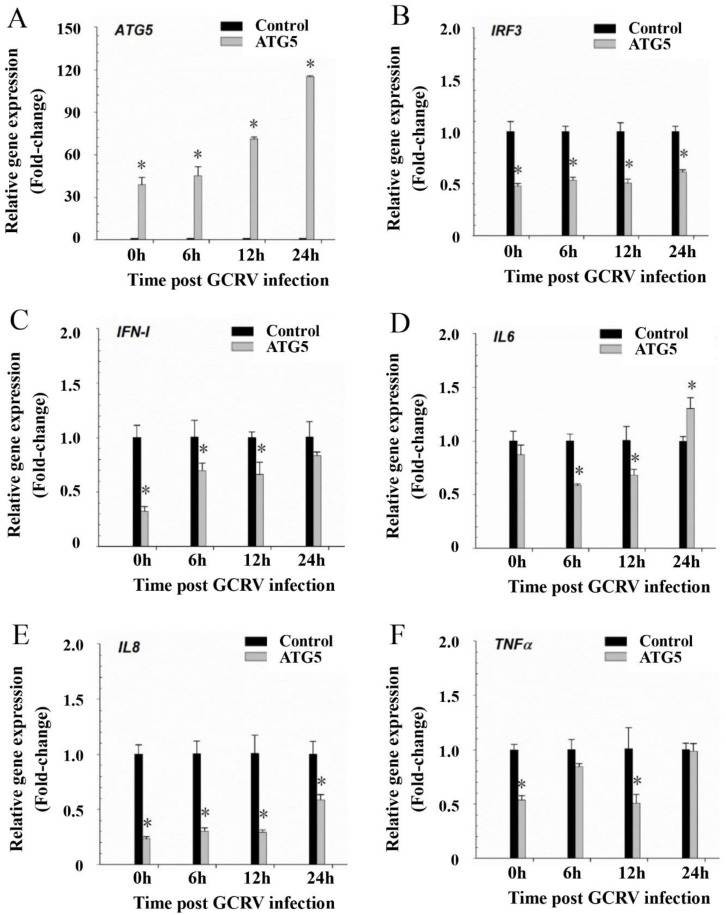
ATG5 attenuated the inflammation response to GCRV stimulation. CIK cells were transfected with CiATG5 or empty vectors for 24 h, followed by GCRV infection. The cells sampled at 0, 6, 12, and 24 h after GCRV infection were used to measure the expression of related genes, (**A**) ATG5, (**B**) IRF3, (**C**) IFN-I, (**D**) IL6, (**E**) IL8, and (**F**) TNFα. One-way ANOVA was used to evaluate the variability between treatment groups and significance differences (*p* < 0.05) in relation to the control group were indicated with an asterisk (*).

GO enrichment analysis showed that the GO terms involved in cell death regulation, including regulation of cell death, regulation of the apoptotic process, regulation of programmed cell death, the apoptotic process, and cell death, were downregulated at 24 h after GCRV infection in CiATG5-overexpressed cells, indicating CiATG5 decreases the cell death response to GCRV infection (Table S4). KEGG enrichment analysis showed that the classic immune response signal pathways, including RIG-I-like receptor, NOD-like receptor, C-type lectin receptor, and Toll-like receptor signaling pathways, necroptosis, and apoptosis were all downregulated at 24 h after GCRV infection in CiATG5-overexpressed cells (Table 2 and Figure 9A–I), indicating there was less inflammatory response in CiATG5 overexpression cells. To verify this conclusion, the mRNA expression levels involved in inflammatory and interferon-related genes were detected by qPCR. As shown in Figure 10, transcriptional expression levels of these genes were all lower in transfected CiATG5 groups. Together, these results indicate that CiATG5 attenuates the acute inflammatory/immune response to GCRV stimulation and then protects cell survival.

### 3.8. CiATG5 Enhanced Integrity of CIK Cells against GCRV Invasion

**Figure 11:**
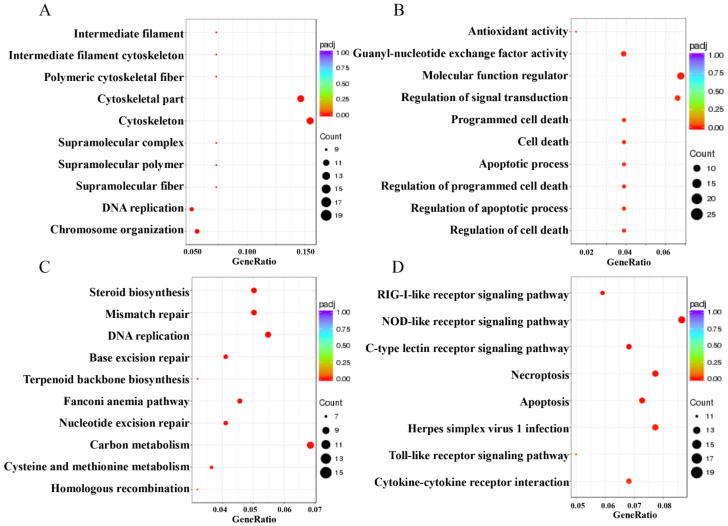
The top enriched GO terms/KEGG pathways of DEGs from 24 h postinfection. The color of the dot represents the level of difference; the size of the dot represents the number of genes that are enriched in the pathway; the abscissa represents the ratio of enriched genes to all genes in the pathway. (**A**) The upregulated GO terms. (**B**) The downregulated GO terms. (**C**) The upregulated KEGG pathways. (**D**) The downregulated KEGG pathways.

**Figure 12:**
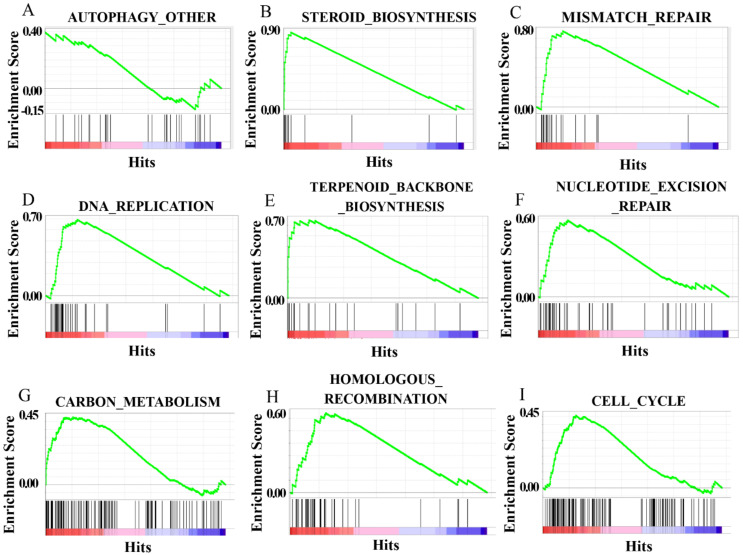
Gene set enrichment analysis (GSEA) of the upregulated genes. The upregulated genes were mainly enriched in autophagy (**A**), steroid biosynthesis (**B**), mismatch repair (**C**), DNA replication (**D**), terpenoid backbone biosynthesis (**E**), nucleotide excision repair (**F**), carbon metabolism (**G**), homologous recombination (**H**), and cell cycle (**I**).

GCRV infection can affect the normal physiological functions of host cells, cause cell damage, and then induce cell death, which has been fully confirmed in the past [41,42]. In this study, GO enrichment analysis showed that the GO terms related to cell components, including DNA and chromosome organization, intermediate filament composition, polymeric cytoskeletal fiber, cytoskeleton, supramolecular complex, and supramolecular fiber, were upregulated in CiATG5-transfected groups compared with the control (Table S4 and Figure 11A). Moreover, KEGG enrichment analysis showed that biosynthesis- and metabolic-related pathways also showed the same trend. In detail, steroid biosynthesis, terpenoid backbone biosynthesis, nucleotide excision repair, carbon metabolism, cysteine and methionine metabolism, homologous recombination, autophagy, and cell cycle were all upregulated in CiATG5-transfected groups (Table 2, Figure 11C and Figure 12A–I). The results indicate that overexpressed CiATG5 strengthens the integrity of CIK cells when GCRV invades.

## 4. Discussion

It has been more than 60 years since autophagy was first observed [43]. Notably, over the past few years, the studies on autophagy have become a top subject of particular scientific interest because of accumulating evidence that indicates that autophagy is closely related to viral infection and replication [13,14,15,16,17,18,19,20,21,22,23]. On the one hand, autophagy plays an irreplaceable role in inflammasome activation and antigen processing for presentation and the cooperation between ATGs and other immune proteins to activate immune pathways to restrict viral infections [5]. On the other hand, viruses employ multiple strategies to avoid the antiviral functions of autophagy and even utilize the double-membrane compartments formed during autophagy to provide a small isolated membrane envelope for viral replication, package, and maturation [18,44]. It seems autophagy functions as a double-edged sword for antiviral infection. Autophagy is a universal cellular process that can be observed in all nucleated cell types, from yeast to man [45]; it also takes place in fish. The first report on fish-virus-induced autophagy was in Atlantic salmon [25]. The result showed that inhibition of autophagosome formation by 3-MA reduced LC3-GFP puncta formation and viral production. In recent years, a growing number of studies on autophagy in aquatic animal cells have been reported; however, there is still limited knowledge in this field, especially in fish. GCRV, a double-stranded RNA (dsRNA) virus, has received special attention over the past few decades because it has caused severe epidemic outbreaks of hemorrhagic disease and resulted in tremendous mortality in grass carp [46]. GCRV infection triggers massive cell death in grass carp during rapid amplification (Figure S1) [47]. Effective antiviral drugs for GCRV have not yet been developed; therefore, it is necessary to understand the pathogenesis of the virus. In this study, we found that GCRV infection promoted autophagy in the spleen (Figure 1B). Furthermore, the dynamic process of autophagy formation was detected in CIK cells. At 6 h after GCRV infection, a few puncta assembled by LC3-II were observed, which indicated that autophagy was activated by GCRV invasion (Figure 2A). Moreover, lysosomes, which are closely related to autophagy, were largely accumulated after GCRV infection (Figure 2D). Our results are very much in agreement with previous data: viral invasion induces the enhancement of autophagy activation and autophagosome formation [13,15,16,17,25].

In fish, autophagy has been confirmed to depend on the mTOR signaling pathway and can be upregulated by autophagy inducer rapamycin and downregulated by autophagy inhibitor 3-MA [48]. Therefore, the most direct way to confirm the effect of autophagy on GCRV replication is to use rapamycin or 3-MA to promote or inhibit autophagy. In this study, we demonstrated that rapamycin pretreatment significantly depressed GCRV replication (Figure 3), while 3-MA greatly increased viral replication (Figure 4). This result was consistent with a recent study, which confirmed that ROS-induced HSP70 stimulates antiviral autophagy in CIK cells [49]. 

In mammals, 40 genes encoding ATG proteins have been identified so far; these ATGs play an important role in regulating the formation of autophagy [5]. In fish, zebrafish autophagy-related genes such as *ATG1*–*ATG9*, *ATG12*, and *ATG16* have been identified. The techniques used for detecting autophagy in mammals, including LC3-II fluorescence tracing and LC3 lipoylation WB, have also been confirmed as effective tools for zebrafish autophagy research, indicating autophagy is a highly conserved process in both mammals and fish [50]. Xia et al. cloned orange-spotted grouper (*Epinephelus coioides*) ATG5 (EcATG5) and confirmed that EcATG5 is a cytoplasmic protein, while our former study showed that CiATG5 is located in both cytoplasm and nucleus; the two results were not consistent with each other, implying ATG5 may function in different roles. In grouper spleen (GS) cells, knockdown of EcATG5 decreased the expression of transcription and protein levels of SGIV and RGNNV genes, while EcATG5 overexpression showed the opposite results [27]. In NT-2 cells (a pluripotent human testicular embryonal carcinoma cell line), ATG5-knockdown using small hairpin RNA (shRNA) enhanced the activation of autophagy and reduced the viral replication of JEV RP-9 [13]. A dominant-negative ATG5 mutant in embryonic stem (ES) cells suppressed autophagic vesicle formation and inhibited HCV infection [14]. In CIK cells, CiATG5 overexpression increased autophagy levels, as evidenced by the increasing number of autophagosomes (Figure 5A–C), and strongly restricted the expression of structural protein genes and nonstructural protein genes of GCRV (Figure 6), which indicates that ATG5-triggered autophagy suppresses GCRV production. The functional distinction of ATG5 and autophagy during virus infection may be attributed to species and virus specificity. Therefore, our data open a door for researchers to develop new antiviral drugs targeting ATG5 and autophagy pathways. Moreover, the further investigation of ATG5-induced autophagy will help the exploration of therapeutic strategies against the virus in teleost fishes.

To clarify the mechanism of the inhibitive effect of CiATG5 on GCRV replication, RNA-seq analysis was performed. Transcriptome analysis showed that autophagy was suppressed when GCRV had just invaded cells, indicating that autophagy might not be good for GCRV replication. When zebrafish embryonic fibroblast line cells were infected with viral hemorrhagic septicemia virus (VHSV), autophagy was rapidly activated and the inhibition of autophagy significantly inhibited viral replication, indicating that autophagy promotes VHSV replication in zebrafish cells [51]. When invaded by pathogens, the innate immune system is quickly activated and induces inflammation to protect the host [52]. Pattern recognition receptors (PRRs) play an indispensable role in sensing microbial components and inducing the expression of inflammatory mediators such as cytokines and type I interferons. However, the innate immune system must be strictly regulated to avoid either insufficient inflammatory responses or excessive inflammatory responses, such as septic shock, autoimmune diseases, and metabolic diseases [5]. Accumulating evidence has shown that the autophagy-dependent degradation system has an important role in regulating inflammatory responses [53,54]. In this study, transcriptome analysis showed that the number of DEGs after GCRV infection was significantly lower in CiATG5 overexpression groups (Figure 7). Further analysis showed that KEGG terms involved in PRRs in CiATG5 overexpression groups, including NOD-like receptor, C-type lectin receptor, RIG-I-like receptor, and the Toll-like receptor signaling pathway, were lower than that of the control at 24 h after GCRV infection. Moreover, GO enrichment analysis showed DNA and chromosome organization, intermediate filament composition, polymeric cytoskeletal fiber, cytoskeleton, supramolecular complex, and supramolecular fiber of CiATG5 overexpression groups were intact compared to the control. These results demonstrate that CiATG5 protects CIK cells from excessive inflammatory responses and cell damage caused by GCRV infection. In human beings, abundant evidence emphasizes that the SARS-CoV-2 infection induces a systemic inflammatory cytokine storm, resulting in myocarditis, arrhythmias, and myocardial damage [55,56,57,58]. Furthermore, identification of the SARS-CoV-2-associated cytokine storm as a potential therapeutic target is supported by the research data [59]. Therefore, ATG5-induced autophagy may be a new therapeutic strategy for cytokine storms.

In conclusion, GCRV-triggered autophagy was confirmed in the study. The results demonstrate that CiATG5 acts as an important autophagy inducer and anti-inflammatory regulator to restrict virus replication. Additionally, our results provide new evidence that autophagy plays an important role in antiviral defense.

## Figures and Tables

**Table 1:** The top enriched Kyoto Encyclopedia of Genes and Genomes (KEGG) pathways of the differentially expressed genes (DEGs).

Comparisons	KEGG Pathways (UP)	KEGG Pathways (DOWN)
H6 vs. HO	Ribosome biogenesis in eukaryotes	Lysosome
	RNA transport	Valine, leucine and isoleucine degradation
	Spliceosome	Other glycan degradation
	Cell cycle	Autophagy—animal
	RNA polymerase	Carbon metabolism
	Protein processing in endoplasmic reticulum	Steroid biosynthesis
	Aminoacyl-tRNA biosynthesis	Fatty acid degradation
	mRNA surveillance pathway	Pyruvate metabolism
	Protein export	mTOR signaling pathway
	Cytosolic DNA-sensing pathway	Glycolysis/Gluconeogenesis
H12 vs. HO	Proteasome	Lysosome
	Protein processing in endoplasmic reticulum	Other glycan degradation
	DNA replication	Biosynthesis of amino acids
	TGF-beta signaling pathway	Carbon metabolism
	Spliceosome	Steroid biosynthesis
	Protein export	mTOR signaling pathway
	Cell cycle	Glycolysis/Gluconeogenesis
	Arachidonic acid metabolism	Aminoacyl-tRNA biosynthesis
	Mismatch repair	Glyoxylate and dicarboxylate metabolism
		Fatty acid degradation
H24 vs. HO	C-type lectin receptor signaling pathway	Oxidative phosphorylation
	RIG-I-like receptor signaling pathway	Carbon metabolism
	MAPK signaling pathway	Valine, leucine and isoleucine degradation
	Toll-like receptor signaling pathway	Ribosome
	NOD-like receptor signaling pathway	Propanoate metabolism
	Foxo signaling pathway	Homologous recombination
	Herpes simplex virus 1 infection	Fatty acid degradation
	Apoptosis	Fatty acid metabolism
	Mitophagy—animal	Peroxisome
	Autophagy—animal	Fanconi anemia pathway

**Table 2:** The top enriched KEGG pathways of the differentially expressed genes.

Comparisons	KEGG Pathways (UP)	KEGG Pathways (DOWN)
ATG5–H0 vs. VT–H0	Carbon metabolism	Ribosome biogenesis in eukaryotes
	Protein processing in endoplasmic reticulum	RNA transport
	Steroid biosynthesis	Aminoacyl-tRNA biosynthesis
	Valine, leucine and isoleucine degradation	RNA polymerase
	Fatty acid metabolism	Spliceosome
	Phagosome	RNA degradation
	Fatty acid degradation	Cytosolic DNA-sensing pathway
ATG5–H6 vs. VT–H6	Cell cycle	Ribosome biogenesis in eukaryotes
	Steroid biosynthesis	Aminoacyl-tRNA biosynthesis
	Lysosome	Mismatch repair
	Apoptosis	RNA transport
	Terpenoid backbone biosynthesis	Nucleotide excision repair
	Progesterone-mediated oocyte maturation	DNA replication
	Fatty acid metabolism	Porphyrin and chlorophyll metabolism
	Oocyte meiosis	Cysteine and methionine metabolism
	Cellular senescence	Glutathione metabolism
		RNA degradation
ATG5–H12 vs. VT–H12	Steroid biosynthesis	Glutathione metabolism
	Terpenoid backbone biosynthesis	Arachidonic acid metabolism
	Cell cycle	Ferroptosis
	Focal adhesion	Oxidative phosphorylation
	Tight junction	Porphyrin and chlorophyll metabolism
	DNA replication	
	AGE-RAGE signaling pathway in diabetic complications	
ATG5–H24 vs. VT–H24	Steroid biosynthesis	RIG-I-like receptor signaling pathway
	Mismatch repair	NOD-like receptor signaling pathway
	DNA replication	C-type lectin receptor signaling pathway
	Base excision repair	Apoptosis
	Terpenoid backbone biosynthesis	Necroptosis
	Fanconi anemia pathway	Herpes simplex virus 1 infection
	Nucleotide excision repair	Toll-like receptor signaling pathway
	Carbon metabolism	Cytokine-cytokine receptor interaction
	Cysteine and methionine metabolism	
	Homologous recombination	

## Supplementary Materials

The following are available online at https://www.mdpi.com/2218-273X/10/9/1296/s1. Figure S1: The quick replication of GCRV after infection in CIK cells. (**A**) GCRV infection caused an obvious cytopathic effect in CIK cells. (**B**) CIK cells were sampled at 6, 12, and 24 h after GCRV infection to measure the expression of NS and VP genes of GCRV, and one-way ANOVA was used to evaluate the variability between treatment groups (*p* < 0.05). Table S1: Summary of sequencing data of CIK cell samples infected with GCRV. Table S2: Summary of sequencing data of ATG5-overexpression CIK cells infected with GCRV. Table S3: The top enriched GO terms of DEGs in CIK cell samples infected with GCRV. Table S4: The top enriched GO terms of DEGs of ATG5-overexpression CIK cells infected with GCRV. Table S5: Primers used in this study.
